# Implementation of community physiotherapy in primary care: one-year results of an on-call physiotherapy service

**DOI:** 10.1186/s40945-023-00176-3

**Published:** 2023-12-14

**Authors:** Matteo Paci, Lapo Bianchi, Elisa Buonandi, Laura Rosiello, Sandra Moretti

**Affiliations:** Dipartimento delle Professioni Tecnico-Sanitarie, Azienda USL Toscana Centro, Florence, Italy

**Keywords:** Primary health care, Referral and consultation, Physical therapy modalities, Program evaluation, Outcome and process assessment

## Abstract

**Background:**

Primary health care systems have a key role in meeting health needs of community, including function. The aim of this paper is to describe the population involved in the Community Physiotherapist project and their health outcomes over a one-year period.

**Methods:**

The Community Physiotherapist is an on-call service which requires a request by general practitioners or medical specialists. Reason for prescription, waiting time for service delivery, diagnostic categories*,* provided intervention, number of interventions and outcomes were recorded for everyone included in the project. Possible differences in characteristics between individuals referred by medical specialists and general practitioners were also investigated.

**Results:**

From January to December 2022, 409 individuals were referred to the Community Physiotherapist pathway. Functional goals were achieved in 79.5% of interventions, without reported adverse events. In most cases physiotherapists provided counselling or caregiver training and 3.3% of individuals needed a full rehabilitation program. The groups of individuals referred by the two types of prescribers showed no significant differences, apart, as expected, from their median age.

**Conclusions:**

The introduction of the Community Physiotherapist model within the primary care setting allows to provide appropriate, effective and safe interventions. Sharing the project among all the health professionals helped to support its appropriateness and effectiveness. Results also indicate that a new organizational model, such as the Community Physiotherapist, will take a long time to be implemented.

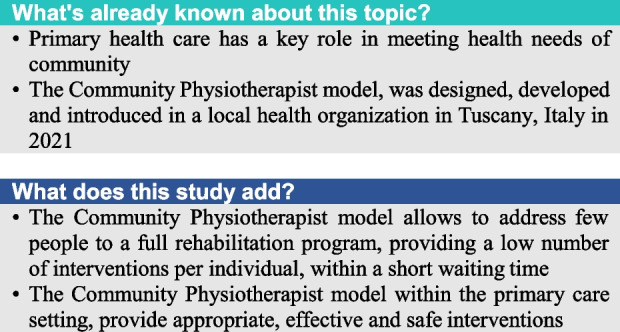



## Introduction

Primary health care (PHC) is a comprehensive, interdisciplinary, patient-centered and community-oriented approach to health care. It is the patient’s first point of entry into the health care system and the continuing focal point for their healthcare needs [1].

Demographic and health changes concur to increase the prevalence of disability or functional decline [2] and it is expected that at least one third of the global population will need rehabilitation at some point over the course of their illness or injury [3].

The key role of PHC in meeting health needs of community was declared by the Alma Ata Conference in 1978 [4] and, more recently, reinforced by the Declaration of Astana in 2018 [5]. Function, one of the most important health indicators, also optimized by rehabilitation, should be included in the primary care system as well [6]. The term “healthcare” generally includes a broad spectrum of determinants incorporating social and environmental needs [7]. The term “community” can also include a wide number of characteristics (e.g., economic conditions, race and ethnicity); geographic location of both population and health facilities, as well as specific health problems are the most frequently described criteria to define health needs [7].

Previous innovative care models of physiotherapy services implementation within PHC have been proposed [8–10], and benefits from both health and economic perspectives, (i.e., reduction of waiting times, improvement of health outcomes, reduction of costs and rates of medication prescribing) have been suggested [11].

Conversely, in the Italian public health system, a physician-based, generally medical-specialist-based, paradigm of care is currently adopted, and physiotherapy interventions are still not integrated within the primary care system, despite being widely recommended in different countries [12, 13].

A wide number of investigations describe models and outcome of direct access, self-referral to or triaging by physiotherapists [14–16], but these models’ implementation is still not allowed by national legislation within the Italian public national health system. In addition, although many qualitative studies report barriers, opportunities and perspectives of physiotherapists and other health professions [9, 17, 18] on the implementation of physiotherapy within PHC, quantitative studies are still lacking and mainly referred to musculoskeletal conditions [8, 12, 13, 19].

From these perspectives, inspired by the Expanded Chronic Care Model and consistently with the Italian National Recovery and Resilience Plan, [19] an innovative rehabilitation service project, the Community Physiotherapist model, was designed, developed and introduced for the first time in Italy in a local health organization in Tuscany in 2021 [20]. The model refers to an on-call physiotherapy service embedded in a multidisciplinary team, including Family and Community Nurse, general practitioner, medical specialist and other health or social professional as needed, where the general practitioner plays a key role as case manager. Preliminary findings showed Community Physiotherapist as an effective and safe model, in terms of achievement of a priori objectives defined by the team and low rate of adverse events, respectively; however, results could be partially affected by organizational adaptations due to peak phase of COVID 19 pandemic.

The aim of this paper is to describe the population involved in the Community Physiotherapist model implementation and report the health outcomes, over a one-year period in a less acute phase of the pandemic.

## Methods

This is an empirical study of the implementation of a project potentially addressed to the whole population managed by all general practitioners working within the local health organization, a territorial health service organized in four functional territorial units.

The development of the model and its implementation phases are described in detail in a previous paper [20].

Briefly, the target population comprised persons having: (i) significant reduction in functional autonomy; (ii) increased care burden or need for caregiver training; (iii) history of falls/risk of falling; or (iv) need for home environment assessment.

The Community Physiotherapist is an on-call service which requires a request by general practitioners or medical specialists, mainly Geriatricians and Internal medicine specialists belonging to GIROT (Gruppo Intervento Rapido Ospedale Territorio), a “hospital-at-home” mobile multidisciplinary team regularly connected to general practitioners [21], currently available only for one functional territorial unit, representing about half of the whole population.

Typical interventions included: (i) provision of counselling to families and caregivers on mobility aids; (ii) strategies to reduce the risk of falls; (iii) support aimed to maintain functional status and (iv) identification of the need for more extensive rehabilitation interventions or for intervention of other team professionals, through specific pathways.

The interventions, provided in the outpatient clinic or individual’s home, were based on a patient-centredness perspective [22], mainly focused on health education, self-management and empowerment aimed at both patient and caregiver, with the possibility of remote access for follow-ups provided through telephone or video calls. The project included a maximum of 4 sessions of about 1 hour each: when more interventions were needed, the conventional rehabilitation pathway was activated.

For each individual included in the project, the following data were recorded: reason for the referral (target population), waiting time for service delivery, diagnostic categories (i.e., neurologic, musculoskeletal or oncologic pathologies, frailty, hypomobility syndrome, comorbidity), setting (clinic or individual’s home), provided intervention (Counselling, review of mobility aids and equipment; full rehabilitation path; caregiver training; falling prevention training or other), number of interventions; and outcome, understood as achievement of functional goals, which was understood as the achievement of functional goals which had been established collaboratively with the general practitioners and medical specialists. Functional goals were tailored to the needs of the person/caregiver and based on reasons for prescription, diagnostic categories and provided interventions*.* Before the intervention starts, goals were also communicated and shared with the person/caregiver.

Data were firstly collected in a local database by physiotherapists involved in the project and at the end of each semester, once anonymized, were sent to a professional study manager for aggregation and analysis.

**Fig. 1:**
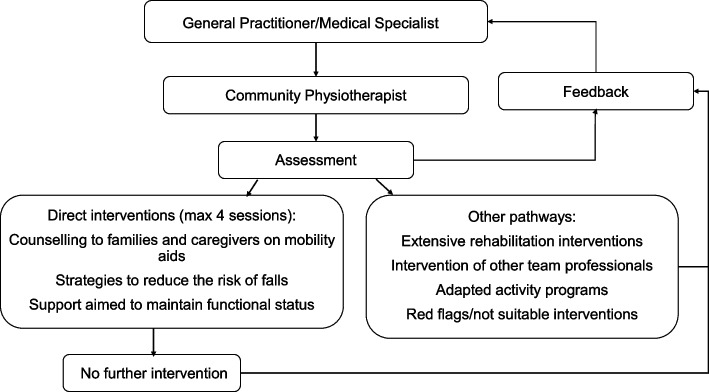
Flow-chart of the Community Physiotherapist pathway

A flow-chart of the pathway is shown in Fig. 1*.*

### Statistical analysis

The normal distribution of the collected continuous data (age and time of intervention) was assessed using the Shapiro-Wilk Test. Since distribution of these variables departed significantly from normality (*W* = .88; *p* < .001 and *W* = .59; *p* < .001, respectively), median and interquartile range (IQR) were used to represent continuous variables. In case of categorial data, frequencies and percentages were used. The number of cases in which functional goals were met was compared among clinical categories and consultancy activation areas, using the chi square test.

In order to investigate possible differences in characteristics between individuals referred by *GIROT* medical specialists and general practitioners (within the territorial unit where it was operating), additional analyses were performed to compare age, waiting time, and number of treatments using the Mann-Whitney test, and diagnostic categories, number of referrals, and setting, using the chi square test. The significance level was set to .05.

Analyses were performed with IBM SPSS Statistics software for Windows (version 20.0; IBM Corp, Armonk, New York, USA).

## Results

From January to December 2022, 409 individuals were referred by general practitioners or medical specialists to the Community Physiotherapist pathway. The median age of included individuals was 85.9 (IQR: 79.8; 90.4) and 165 of them were males. The service was requested by GIROT specialists for 99 (24.2%) individuals, while the others were forwarded by general practitioners.

Healthcare needs of patients included in the sample are described in Table 1.

**Table 1:** Main clinical categories and consultancy activation areas (*N* = 409)

Main clinical categories	*N* (%)	Goals achieved (%)
Multi-pathological	189 (46.2)	162 (85.7)
Long-term immobilization consequences	95 (23.3)	81 (85.3)
Neurological	59 (14.4)	47 (79.7)
Frail patient	25 (6.1)	20 (80.0)
Musculoskeletal	37 (9.0)	30 (81.1)
Oncological	4 (1.0)	2 (50.0)
		*p* = .557
**Consultancy activation areas**		
Significant reduction in functional autonomy	290 (70.9)	240 (82.8)
Increased care burden or need for caregiver training	81 (19.8)	72 (88.9)
History of falls/risk of falling	28 (6.8)	22 (78.6)
Home environment assessment	10 (2.5)	8 (80.0)
		*p* = .077

Interventions were activated within a median of 7.0 days (IQR: 5.0; 12.0); The greater part of interventions were provided at patient’s home (*n* = 396; 97%); Within those interventions, both in-person and remote sessions per patient were 1.0 (IQR: 1.0; 2.0).

Functional goals were achieved in 79.5% (*n* = 341) of interventions, without significant differences among clinical categories and consultancy activation areas (Table 1)*.*

Adverse events were not reported; in most cases physiotherapists provided counselling or caregiver training, other pathways were indicated to 21 individuals, out of whom 14 (3.3%) needed of a full rehabilitation program, while adapted activity programs [23] were suggested to the others (Table 2).

**Table 2:** Provided interventions (*N* = 409)

Provided interventions	*n* (%)
Counselling	244 (59.7)
Caregiver training	106 (25.9)
Review of mobility aids and equipment	32 (7.8)
Other pathways	21 (5.1)
Falling prevention training	6 (1.5)

Within the territorial unit where GIROT was operating, 267 individuals were included in the project. No differences were found between GIROT and general practitioners in terms of waiting time (median = 7; IQR:4–13 and median = 9; IQR: 6–14, respectively; *p* = .121), and number of provided treatments (median = 3; IQR:2–3 for both groups; *p* = 368). Differently, GIROT referred older individuals (median = 87.9; IQR:82.7–92.1 versus median = 85.7; IQR: 79.8–89.8; *p* = .014). No differences were found in terms of main clinical categories, setting and goals achievement (Table 3).

**Table 3:** Differences between GIROT and Geral practitioners in main clinical categories and intervention setting

Variables		GIROT (*n* = 98)	Geral practitioners (*n* = 169)	*P* value
Main clinical categories (%)				.058
	Multi-pathological	33 (34)	50 (30)	
	Long-term immobilization consequences	38 (39)	43 (25)	
	Neurological	15 (15)	32 (19)	
	Frail patient	5 (5)	16 (9)	
	Musculoskeletal	7 (7)	25 (15)	
	Oncological	0 (0)	3 (2)	
Setting (home/clinic)		98/0	167/2	.282
Goals achieved (%)		78 (80)	142 (84)	.324

## Discussion

This report describes a cohort of individuals involved in the Community Physiotherapist project in 2022. Preliminary findings, related to 9 months of 2021 were recently published [20]. When compared to the previous cohort, a higher number of consults was requested in 2022, although the reference periods are different (9 versus 12 months), suggesting a larger dissemination of the project among general practitioners or medical specialists and their higher participation. In addition, by keeping a low number of interventions per individual, the waiting time was furthered reduced and fewer people needed a full rehabilitation program. Finally, no adverse events were reported, as in the previous cohort.

The function goals, which had been established collaboratively by patients/caregivers, prescribers and physiotherapists, were achieved for a high number of interventions, slightly greater than those reported in the previous investigation. This issue can be considered as an index of appropriateness for both consultancy activation and intervention efficacy.

Those findings appear to be relevant, since the speed of service delivery, the reduction of the burden on the rehabilitation services and the low number of interventions make the Community Physiotherapist pathway a flexible, effective, and resilient model to contribute to prevention for chronic conditions exacerbation, meeting the health needs of the community.

It should be highlighted that the comparison with the cohort 2021 could be partially affected by organizational limits due to COVID 19 pandemic. Anyway, the flexibility and resilience of the model is further supported by the promising findings of the model implementation during the acute phase of the COVID 19 pandemic.

The safety and the efficacy of interventions was also made possible thanks to close collaboration with general practitioners or medical specialists. The effectiveness of interventions was assessed using the goal achievement as a proxy, since clinical outcomes and interventions would have been too heterogeneous. Since physiotherapists worked more closely with geriatricians than general practitioners within this health organization before the start of the project, differences between the characteristics of individuals referred by the two types of prescribers might have been expected. Instead, no significant differences were found, apart from the median age, as expected, suggesting that sharing with general practitioners about rational and aim of the project was effective.

The sample involved in the Community Physiotherapist pathway meets the changing health needs of the ageing population, which is the target of calls for development of health services innovative organizations recommended by international [24] and national [19] initiatives.

In fact, the mean age of individuals included in the sample was greater than 80 years and more than two thirds of interventions were addressed to individuals with complex needs (multiple pathologies and long-term immobilization consequences) who had significant reduction in functional autonomy.

A relevant part of interventions was addressed to reduce care burden, a key issue in the management of older adults with chronic illness [25].

Sharing the project among physiotherapists, nurses, geriatricians and internal medicine doctors as well as a specific training addressed to physiotherapists [20] helped to support appropriateness and effectiveness. At the same time, clear structural characteristics of the organization and supportive leadership allowed to reinforce efficiency and adherence to the project. These issues are shown to be key elements to make an organizational change possible in the field of chronic and complex diseases [26, 27].

Telephone follow-up after at-home interventions has been shown to be as effective as in-person treatment of adults with chronic health conditions [28] as well as less costly [29]. Therefore, although training sessions mainly addressed supervision, some time was directed towards monitoring adherence to treatment or to suggest strategies remotely by telephone or video calls.

Previous experiences on implementation of physiotherapy within PHC were usually referred to musculoskeletal conditions [8, 12, 13, 30], while the Community Physiotherapist model was addressed to a wider and more complex population, including different clinical categories. Other studies reported promising results about models based on direct access, self-referral to or triaging by physiotherapists [14–16] which cannot be implemented within the Italian public health system context. In light of this scenario, the model seems to be an innovative and feasible way to achieve a trade-off between the need to include physiotherapy within PHC and the rules of the Italian health care system.

Although the project started in 2021, some limitations must be reported for its implementation. The information and communication campaign should be reinforced, since still only a part of general practitioners requested the service; in addition, it is possible that cultural resistances may have occurred, since the implementation of a new organizational model frequently requires a cultural challenge and time to fully develop.

For this reason, further educational and communication initiatives should be implemented including all actors involved in the pathway. Qualitative research could help to further investigate barriers and facilitators perceived by all healthcare professions to the model implementation.

Finally, the project is addressed only to the tertiary prevention at the present time. Since PHC is a privileged area to also provide both primary and secondary prevention, the role of Community Physiotherapist should be expanded on these additional levels.

## Conclusions

Findings from this investigation confirm that the introduction of the Community Physiotherapist model within the primary care setting, combining models recommended in the literature [6], allows physiotherapists to provide appropriate, effective and safe interventions. However, long time seems to be requested to implement a new organizational model, such as the Community Physiotherapist. The experiences of physiotherapists, general practitioners and medical specialists on barriers to, and facilitators for, the implementation of the Community Physiotherapy model of care, should be explored and the cost/benefit ratio of the model implementation should also be analysed.

## Data Availability

The datasets used and/or analysed during the current study are available from the corresponding author on reasonable request.

## Abbreviations

PHC: Primary Health Care
GIROT: Gruppo Intervento Rapido Ospedale Territorio
IQR: interquartile range
